# History Repeats Itself: The Relevance of Historical Pandemics to the Medical School Curriculum

**DOI:** 10.1177/23821205231210629

**Published:** 2023-11-09

**Authors:** M Jones, S Quenby, J Odendaal

**Affiliations:** 1 Division of Biomedical Sciences, Clinical Sciences Research Laboratories, 12212 Warwick Medical School, University of Warwick, Coventry, UK; 2 2708 University Hospitals Coventry & Warwickshire, Coventry, UK

**Keywords:** medical education, pandemic influenza, curriculum

## Abstract

**Introduction:**

The dramatic global impact of the coronavirus pandemic has increased consideration on epidemiological progressions of pandemics. Measures implemented to reduce viral transmission have been largely historical, comparable in nature with the 1918 and 2009 influenza pandemics, demonstrating the importance of clinicians’ awareness on historical pandemics. Despite this, literature suggests medical students’ knowledge on previous pandemics is poor.

**Objectives:**

This study aims to gather stakeholder information from UK medical students on the importance of including the history of pandemics in the medical school curriculum.

**Methods:**

A cross-sectional cohort study conducted via a mixed question type online survey was distributed to all UK medical schools to explore stakeholder views. Grounded theory emergent coding was used to generate themes to free-text answers and SPSS and Excel were used to analyse quantitative data using pivot tables and Fishers exact tests.

**Results:**

Two hundred and forty-one students consented to take part from eight medical schools in the UK with 98% of these students completing the questionnaire. 34% of students reported having teaching on pandemics with 78% of students stating it would be beneficial. Knowledge was poor with 5.7% of students achieving 100% on knowledge-based questions. 72% of students believed that learning about the history of medicine would be beneficial with 87% of these students referring to ‘benefiting (the) future’ in their answers. Additionally, 79% of students thought it would be beneficial to learn about historical pandemics with reference to the current COVID-19 pandemic.

**Conclusion:**

To date, this is the only UK based study assessing stakeholders’ views on including the history of pandemics in the medical school curriculum. Our findings demonstrate that medical students wish to have more historical content included in their degree to better prepare tomorrow's doctors for situations that may occur when history repeats itself.

## Introduction

Historically, infectious disease has represented a stark mortality burden. Although this has eased over time, infectious diseases still accounted for 7% of deaths in 2017.^1^ Interspersed across history are waves of infectious pandemics such as the plague of Justinian in the sixth century, the black death in the fourteenth century, the 1918 H1N1 influenza pandemic, the 2009 influenza pandemic and more recently COVID-19.^2-4^ The 1918 Influenza pandemic was the most severe pandemic to date, infecting a third of the world's population and killing an estimated 50 million people worldwide, surpassing the number killed in the First World War.^2^ Nevertheless, the adage ‘history repeats itself’ has consistently proved its correctness. The present coronavirus pandemic led people to turn to previous pandemics for guidance.^5,6^ Indeed, not only does the COVID-19 pandemic and 1918 influenza pandemic follow similar epidemiologic curves but the epidemiological curves between the 2009 influenza pandemic and the COVID-19 pandemic share similarities.^7,8^

The spectre of pandemics has long haunted epidemiologists, with an ever-increasing population density, the ability to travel and climate change, leading to concerns about rapid and wide transmissions - concerns that have been borne out in the present coronavirus pandemic. On the other hand, advances in technology since the 1918 H1N1 Influenza pandemic has allowed us to learn more about the pathophysiology, epidemiology, and potential treatment options of viruses such as H1N1 Influenza.^5,9^

Public health measures employed to reduce viral transmission have been largely historical in nature, with the 1918 pandemic guiding initial coronavirus contingency planning. Such comparisons can be directly seen; in 1918 social distancing was introduced, schools and cinemas were shut, and fines were given out for not wearing protective clothing or bulk buying food items.^10^ Similarly, in 2009 measures, such as school closures were implemented, aiming to reduce transmission but research suggested that these were implemented too late.^11^ In 2009 once schools reopened there was a large second wave of cases particularly affecting children.^12^ Similar methods aiming to reduce levels of infection were implemented again in 2020 when COVID-19 spread around the world and like in 2009, the reopening of schools in September 2020 caused a significant increase in cases particularly affecting children.^13^ Not only was Covid-19 similar in epidemiology to the 1918 and 2009 influenza pandemics, but similarities were striking in the way that it affected patients and the proportion of admissions to intensive care.^4^ Thus, further highlighting the importance of teaching and facilitating the understanding of previous pandemics to future doctors.

Several studies have assessed medical students’ understanding of the H1N1 1918 Influenza pandemic and infection prevention methods.^14,15^ In 2007, a study carried out in Canada, demonstrated students had gaps in their knowledge relating to the 1918 Influenza pandemic. Additionally, 28% of students incorrectly believed that antibiotics could treat viral infections and 46.1% of students incorrectly identified the route of transmission.^16^ A similar study performed in China in 2017 found less than 50% of students correctly answering questions on transmission, influenza symptoms and high-risk groups.^17^ These studies highlight the lack of knowledge on pandemics among medical students and further demonstrate the need to include teaching on historical pandemics in the medical school curriculum.

The history of medicine provides an understanding and context of where interventions have worked or errors made.^18,19^ These comparisons and their direct clinical implications between pandemics demonstrate the importance of clinicians’ awareness of historical pandemics.^20^ Doctors and medical staff understandably play a huge role in pandemic care. Questions have been raised since COVID-19 on how much a failure to recognise and understand infection rates, ICU admissions and fatalities based on previous pandemic data led to an increased morbidity and mortality rate during COVID-19.^21^ If there was a greater understanding of pandemic epidemiology, infection rates and morbidity would less potentially erroneous decisions have been made?

The H1N1 pandemic led to advances in infection prevention and pandemic management. Inclusion of previous pandemic in medical education is important to facilitate the understanding of pandemic management.^22^ It also provides a glimpse into the relevance of the history of medicine within the modern medical school curriculum. The condensed nature of many medical school programmes, particularly graduate entry programs in the United Kingdom (UK), means that the curriculum included has to be both concise and fit with the learning outcomes set by the General Medical Council.^23^ The recent outbreak of COVID-19 has raised many questions surrounding medical education, including what role medical students should play in a pandemic and the effects of a pandemic on medical education (10-12).

To date, no study has assessed the knowledge of UK medical students on the 1918 H1N1 Influenza pandemic or assessed medical students’ views on the inclusion of the history of medical pandemics in the medical school curriculum. Given the adage, history repeats itself, this project will assess knowledge of UK medical students on pandemics and related infection control. Further objectives include to establish the current level of teaching on the history of pandemics and gather stakeholder opinions on the role of the medical history of pandemics and the inclusion of other areas of the history of medicine within the medical school curriculum.

## Methods

### Ethical approval

Ethical approval for completion of this study was gained from the University of Warwick Biomedical and Scientific Research Ethics Committee (BSREC 154/19-20). Participation was voluntary and students were informed that responses would not be used as part of their medical degree or influence the results of their degree. Informed consent was gained at the time of participation.

### Study design

A cross-sectional national cohort study delivered via an online survey.

### Questionnaire design

A 21-item questionnaire was devised by the research team to assess the role of medical history within the curriculum. Questions comprised a mixture of styles including multiple choice, open-ended text responses and 5-point Likert-type questions. Questions were designed based on the study aims to ensure capture of key information. The survey was created based on previously published works in the topic area for consistency and to improve comparability across literature.^16,17^ The questionnaire was, therefore, content validated through comparison with the previously published work.^16,17^ Ensuring alignment of published work allowed content validation via proxy expert consensus, it additionally ensured comparability across the literature on the topic. In order to assess questionnaire reliability Cronbach's alpha value was calculated for the survey. Cronbach's alpha value for the 2 5-point Likert-type questions was 0.76 (questions 11 and 12 in the Supplemental Material –Table 1 Copy of Questionnaire). Cronbach's alpha was also calculated for the multiple-choice questions – (questions 6,7,8 and 9 in the Supplemental Material – Table 1 Copy of Questionnaire) and was 0.33. Note this increased with item deletion: 0.44 if question 6 was deleted, 0.74 if question 7 was deleted, 0.69 if question 8 was deleted and 0.74 if question 9 was deleted. Questions were clustered across three themes including: student demographics, knowledge of the H1N1 1918 influenza pandemic and infection control and views on the history of medicine within the medical school curriculum.

**Table 1.** Further analysis of question ‘what is the difference between an epidemic and a pandemic?” (n = 210).

Variable		Correct Answer (n=)	Incorrect Answer (n=)	Value	P-Value
**4-year programme**	Yes	35	1	22.850*	**0**.**001**
	No	169	5S		
**% of the way through the Medicine Course**	100	18	0	31.544*	**0**.**009**
	80	22	6		
	75	4	1		
	70	1	0		
	60	20	0		
	50	18	0		
	40	41	1		
	25	11	0		
	20	69	4		
**Previous teaching on history of pandemics**	Yes	71	0	215.185	**0**.**001**
	No	133	6		
**Previous teaching on the 1918 H1N1 Influenza pandemic**	Yes	33	0	213.163	**0**.**001**
	No	171	6		

Significant P-values are indicated in bold. Values marked by * indicate Fisher Exact test was performed.

The questionnaire was formulated and completed within the Qualtrics Experience Management Platform (Qualtrics International Inc, Utah, U.S.). A full copy of the questionnaire is included in Supplemental Material – Table 1 Copy of Questionnaire.

### Participants

All current medical students studying within a UK medical school at the time of the study were eligible for inclusion. In addition, final year students who graduated early to assist with the coronavirus pandemic were also eligible. All year groups were invited. No power calculation was performed for estimation of sample size selected for the study as all UK medical students were invited. Previous work in this area has been unpowered but demonstrated thematic data saturation at a low sample size.^16,17^

Due to the outbreak of COVID-19, the survey was distributed via an online format only to comply with UK Government regulations at the time. All responses collected were anonymous and no personal identifiers were collected. Consent was gained at the time of questionnaire completion.

### Recruitment

Undergraduate administrators were contacted at all UK medical schools with study details provided including study aims, a link to the survey, a participant information leaflet and consent form. A request was made for inclusion of the study within student cascade emails. Given ethical concerns raised around student overload during the coronavirus pandemic more active recruitment strategies were not pursued. The survey was open from the 18th of December 2020 to the 31st of March 2021.

## Statistical analyses

Descriptive statistics of population demographics including medical school attended, type of course (graduate/undergraduate) and year of study were performed using Microsoft Excel (Microsoft Excel for Mac Version 16.48). Data on year of study was normalised as percentage course completion to allow better between group comparison, given differing course length, with percentage course completion equivalent to year of study divided by course length. Statistical analysis was performed using SPSS statistical software (IBM SPSS Statistics Version 27). Statistical comparison was undertaken for knowledge-based questionnaires. Comparison was performed based on key demographic features including graduate versus undergraduate program, percentage course completion and previous exposure to history of medicine teaching. These were selected to inform firstly against potential confounders but then further to assess the effect of previous history of medicine teaching exposure. Comparison of responses was performed using a Chi-squared test. Where a value within a table was less than five, Fishers exact tests were performed. A significance value of P = <0.05 was used.

A grounded theory emergent coding thematic analysis was undertaken on free text responses using Quirkos (Quirkos (CADAS) for MacOS version 2.4.1). Coding was performed by a single-reviewer following collation of free-text responses and analysed independently of the demographic and knowledge-based questions. Emergent themes were coded, and a second look was performed to ensure all themes that emerged were captured across the responses. Emergent themes were assessed against a grounded theory framework seeking to establish theory on the use of history of medicine teaching within the medical school curricula. This method was selected as it did not pre-suppose the views of the respondents while still allowing these to be assessed against the project aims.

### Reporting

This study is reported in line with the Strengthening of the Reporting of Observational Studies Epidemiology (STROBE) Statement.^24^ A copy of the STROBE statement can be seen in Supplemental material STROBE checklist.

### Funding

No funding was received for the completion of this project.

## Results

Between 11^th^ December 2020 and 31^st^ March 2021 241 responses were collected and all students consented for their data to be used in this study.

### Medical demographics

237 respondents provided completed answers relating to their medical school demographics. Responses were received from 8 medical schools. 4 responses were incomplete. 42 students were on a 4 year degree, 146 students were on a 5 year degree and 52 students were on a 6 year degree.

**Figure 1.**
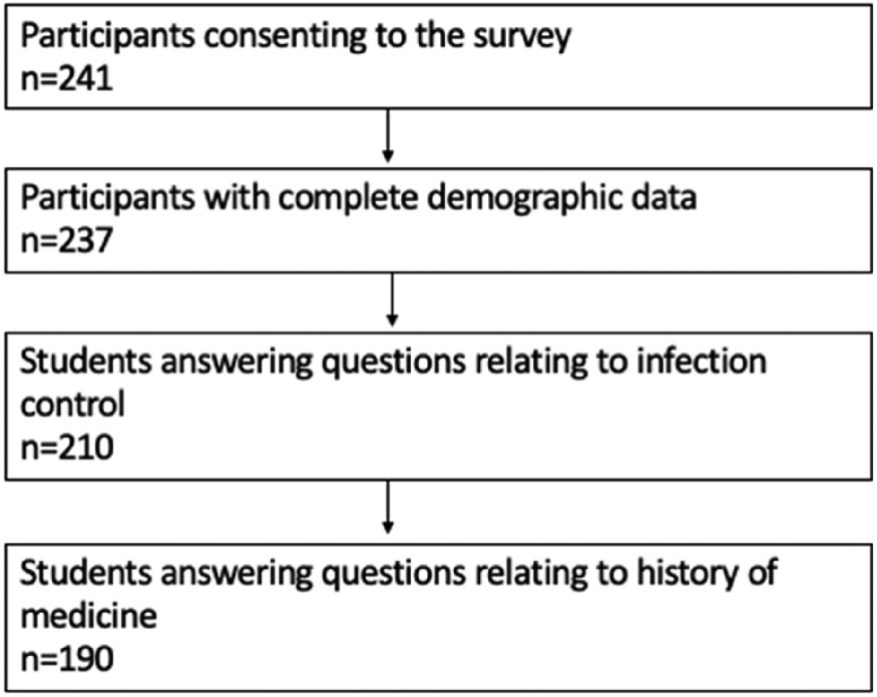
Number of students completing the questionnaire at each section. The questionnaire is split broadly into three sections 1. Student demographics 2. Knowledge of the H1N1 1918 influenza pandemic and infection control 3. Views on the history of medicine in the medical school curriculum. A full copy of the questionnaire is attached in the document titled “Supplemental Material – Table 1 Copy of Questionnaire”.

Figure 1 demonstrates the numbers of participants included at each step of analysis. 17.8% of students were on a 4-year graduate medical programme. No students who completed the survey had recently graduated to help with the ongoing coronavirus pandemic. Course length varied from 4 years to 6 years with most students being on a 5-year programme (n = 146). Median year of study at time of completion of the study was 2 and majority of students were 20% of the way through their medical degree (n = 81).

## Knowledge of the H1N1 1918 influenza pandemic and infection control

### Previous teaching

**Figure 2.**
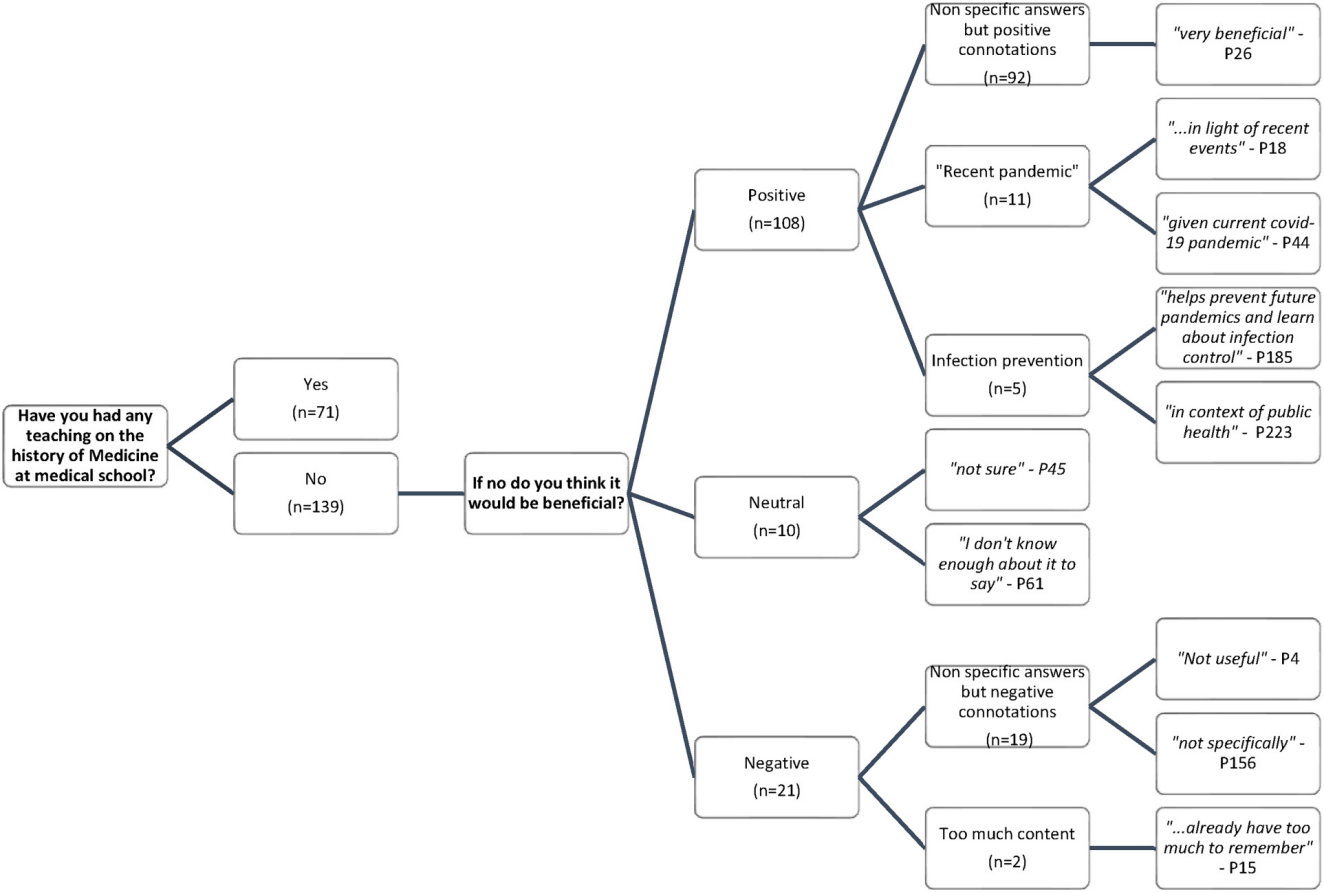
Frequency of answers and themes generated from free text answers relating to the inclusion of the history of medicine at medical school.

34% of respondents reported previous teaching on the history of pandemics (71/210). Of the remaining 139 students that answered no, 78% (108/139) displayed a positive stance to inclusion of the history of pandemics within the curriculum. Distribution of the themes generated from free-text responses is shown in Figure 2.

Where reasons for a positive response were given, these centred on the recent pandemic including “*in light of recent events”* and “*given the current COVID-19 pandemic*”. Additionally, 5 students reported it being beneficial in terms of infection control and prevention. 10 students reported a neutral response with terms including “*maybe”, “not sure”* and “*perhaps”.* 21/122 students’ (9%) displayed a negative stance to inclusion of the history of pandemics with 2 students explanations surrounding content of medical school exams for example, “*…already have too much to remember. This would be extra examinable content*”.

**Figure 3.**
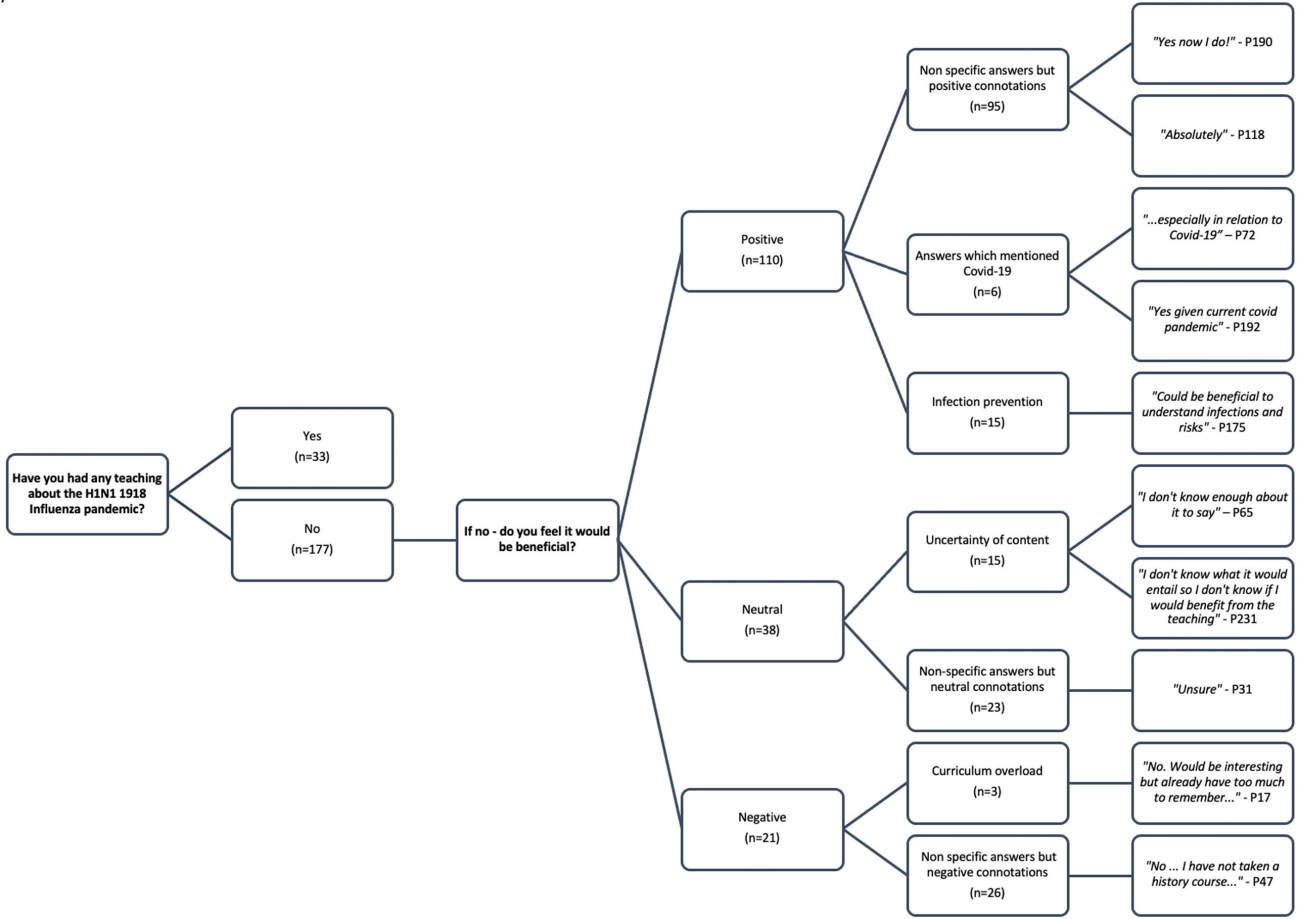
Frequency of answers and themes generated from free text responses in relation to including the 1918 H1N1 influenza pandemic in the medical school curriculum.

33/210 (15%) of students reported specific teaching on the 1918 H1N1 Influenza Pandemic at Medical school and this data is shown in Figure 3.

Of the remaining 177 students responding no, 62% (110/177) displayed a positive desire to have this included within the curriculum with 9 students commenting that it would be useful from a public health perspective and 6 students mentioning COVID-19 in their answers. 15/167 (9%) of student's answers centred on themes of uncertainty on content “*I don’t know what it would entail so I don’t know if I would benefit…”* and “*Depends on how they teach it and what its learning objectives are”.* Finally, 29/167 (17%) students showed a negative stance with 3 students being grouped into the theme - curriculum overload.

### Knowledge on pandemics and infection control

Overall, knowledge was poor, with only 12/210 (5.7%) students answering all 4 questions correctly and median number of correctly answered questions was 2. A full copy of the multiple choice questions used to ascertain knowledge is included in Supplemental Material – Table 1 Copy of Questionnaire.

97% (204/210) of students correctly identified the difference between a pandemic and an epidemic. Students were more likely to correctly answer if they had had previous teaching on the history of pandemics (100% vs 96% P = 0.001) (Table 1).

76/210 (36%) of students correctly identified all 3 methods of transmission of influenza spread (close contact with infected person, coughs and sneezes and contact with infected animals). 30/210 (14%) of students incorrectly identified that influenza can be spread via blood transfusions and 36/210 (17%) of students incorrectly identified that influenza can be spread via sexual contact. No significant difference with previous teaching was seen. However, students were more likely to correctly answer how influenza can spread if they were closer to the start of their degree (43% vs 56% P = 0.009) (Table 2).

**Table 2.** Further analysis of question select all correct answers for how influenza can be spread (n = 210).

Variable		Correct Answer n=	Incorrect Answer n=	Value	P-Value
4-year programme	Yes	13	23	43.184*	0.072
	No	63	111		
% of the way through the Medicine Course	100	7	11	148.403*	0.048
	80	10	22		
	75	2	2		
	70	0	1		
	60	9	11		
	50	4	14		
	40	17	25		
	25	4	7		
	20	23	50		
Previous teaching on history of pandemics	Yes	24	47	50.520*	0.005
	No	52	87		
Previous teaching on the 1918 H1N1 Influenza pandemic	Yes	11	22	45.925	0.035
	No	65	112		

Correct Answer is referring to all 3 methods of transmission being correctly identified (close contact with infected person, coughs and sneezes and contact with infected animals). Incorrect answer refers to all other combinations of answers. Significant P-values are indicated in bold. Values marked by * indicate Fisher Exact test was performed.

Limited knowledge on reduction of transmission in viral pandemics was seen with only 53/210 (25%) of students correctly identifying all 7 methods (social distancing, covering nose and mouth when coughing/sneezing, hand washing, staying home, wearing protective clothing when in public places, antiviral drugs and vaccinations). No significant difference was seen by year of medical school (P = 0.195) (Table 3). Students were more likely to answer correctly if they had not had previous teaching on the history of pandemics (21% vs 27%; p = 0.013).

**Table 3.** Further analysis of question select all correct answers for how influenza/viral pandemic can be prevented.

Variable		Correct Answer n=	Incorrect Answer n=	Value	P-Value
**4-year programme**	Yes	10	26	99.107*	0.107
	No	43	131		
**% of the way through the Medicine Course**	100	2	16	379.882*	0.195
	80	4	69		
	75	3	8		
	70	0	42		
	60	6	12		
	50	2	18		
	40	7	35		
	25	4	7		
	20	25	48		
**Previous teaching on history of pandemics**	Yes	15	56	103.846*	**0**.**013**
	No	38	101		
**Previous teaching on the 1918 H1N1 Influenza pandemic**	Yes	8	25	97.207*	0.090
	No	45	132		

Correct Answer is referring to all 7 methods of being correctly identified (social distancing, covering nose and mouth when coughing/sneezing, hand washing, staying home, wearing protective clothing when in public places, antiviral drugs and vaccinations). Incorrect answer refers to all other combinations of answers. Significant P-values are indicated in bold. Values marked by * indicate Fisher Exact test was performed.

82/210 (39%) of students correctly identified treatment options for influenza. Students were more likely to answer correctly if they were towards the start of their medical degree (P = 0.030) (Table 4). 44% of students with previous teaching on the history of pandemics answered correctly compared to 37% without (P = 0.051) (Table 4).

**Table 4.** Further analysis of question select all correct answers for how influenza/viral pandemic can be treated?”

Variable		Correct Answer n=	Incorrect Answer n=	Value	P-Value
**4-year programme**	Yes	12	24	71.174*	**0**.**027**
	No	70	104		
**% of the way through the Medicine Course**	100	6	12	259.811*	**0**.**030**
	80	8	15		
	75	1	3		
	70	0	1		
	60	12	8		
	50	7	11		
	40	13	29		
	25	5	6		
	20	30	43		
**Previous teaching on history of pandemics**	Yes	31	40	67.681*	0.051
	No	51	88		
**Previous teaching on the 1918 H1N1 Influenza pandemic**	Yes	15	18	67.602*	0.078
	No	67	110		

Correct Answer is referring to both methods of being correctly identified (bedrest and antivirals). Incorrect answer refers to all other combinations of answers. Significant P-values are indicated in bold. Values marked by * indicate Fisher Exact test was performed.

### History of medicine

**Figure 4.**
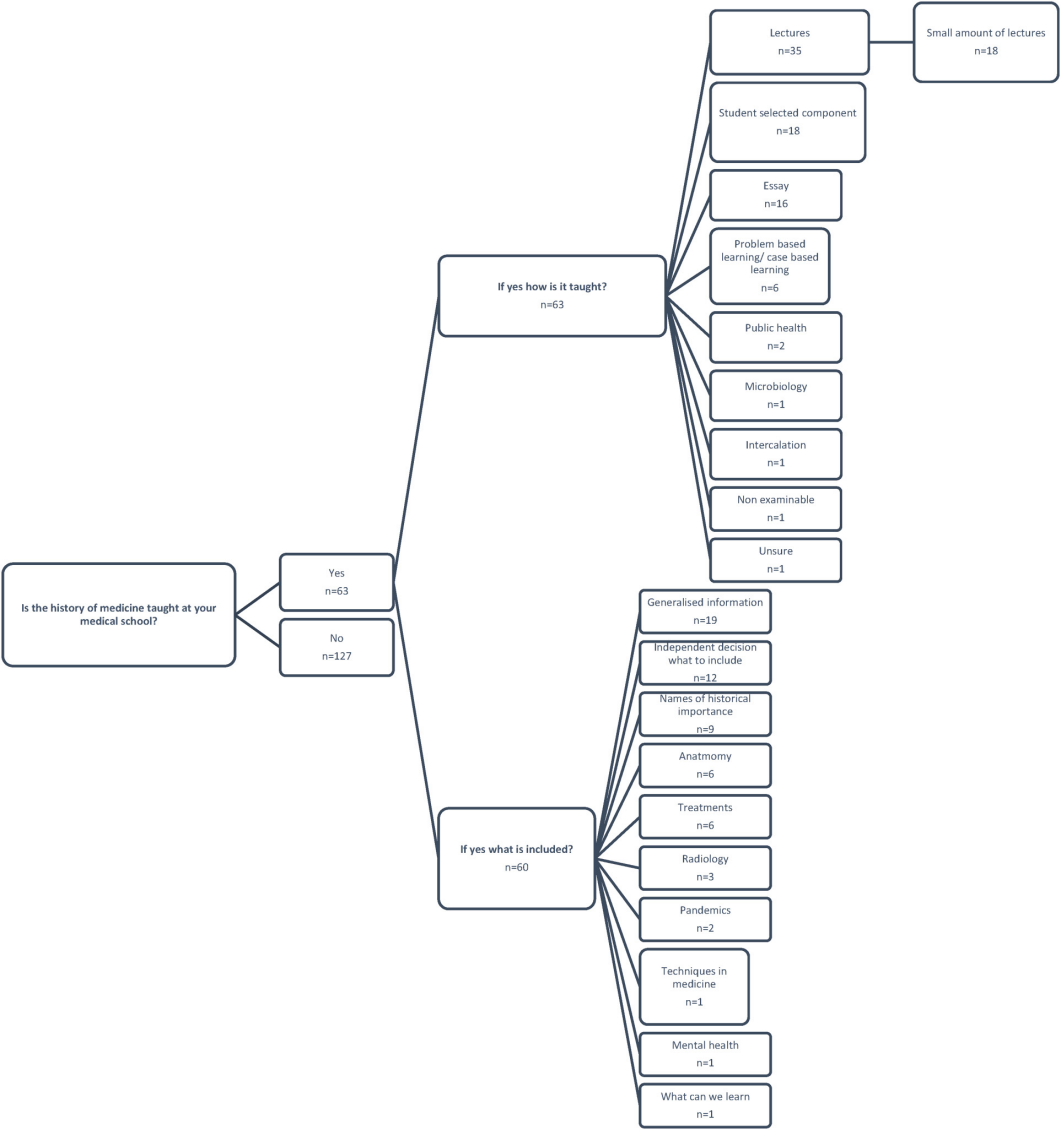
Frequency of answers and themes generated from free text responses in relation to whether the history of medicine in included in the curriculum, what this includes and how it is taught.

Only 33% (63/190) of students reported that the history of Medicine is taught at their medical school (Figure 4).

Of those reporting teaching over half (56% - 35/63) reported this being via lectures with the remainder reporting being taught by essays, problem-based learning, or student selected components. 29% of comments were grouped around the emergent theme of brief engagement with the topic including “*very limited amounts”*, “*very briefly”* and “*brief relevant points at the start of a lecture”*. Most content was coded around the theme of generalised, “*general epidemiology*”, “*pretty much everything”*, “*a wide range of topics”*. 12 students reported having choice over the area that they study, and 6 students reported learning the history of Medicine in relation to Anatomy. 4 students reported pandemics being taught with one commenting “*a bit on famous pandemics like HIV, H1N1, Swine Flu”*. Additionally, only one student commented on the impact of learning about the history of Medicine with regards to current care: “*what we can learn from our history to provide more ethical care now”*.

**Figure 5.**
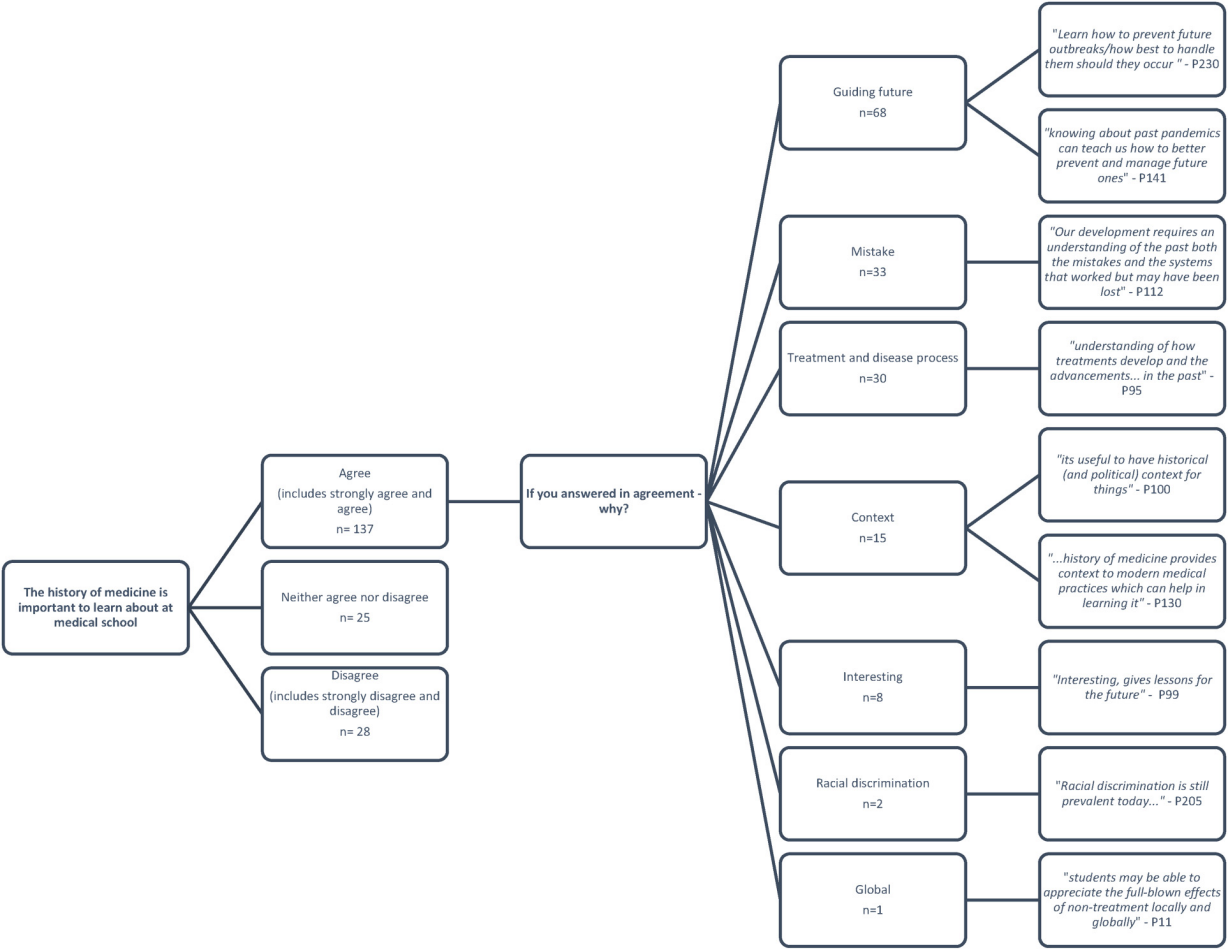
Frequency of answers and themes generated from free text responses in relation to the statement: The history of medicine is important to learn about at medical school.

72% (137/190) of students were positive about the importance of learning history of medicine as demonstrated in Figure 5.

Only 15% (28/190) of students answered disagree. Those with a positive response (87%) included themes such as helping to guide future practice: *“To better prepare students for potentially needing to work in pandemic conditions in the future”* and “*History repeats itself so we must learn from it”*. 24% of students’ responses were themed around mistake avoidance. Additionally, one student commented, “*I only know as much as I do due to my essay title, but I think it would have been important for everyone… By learning history of Medicine, we can learn from previous mistakes…”.*

Overall, students were positive (79% 150/190) about the benefit of learning around the H1N1 pandemic given the Covid-19 pandemic. Only 7% of students answered strongly disagree or somewhat disagree and the remainder (14%) answering neither agree nor disagree.

**Figure 6.**
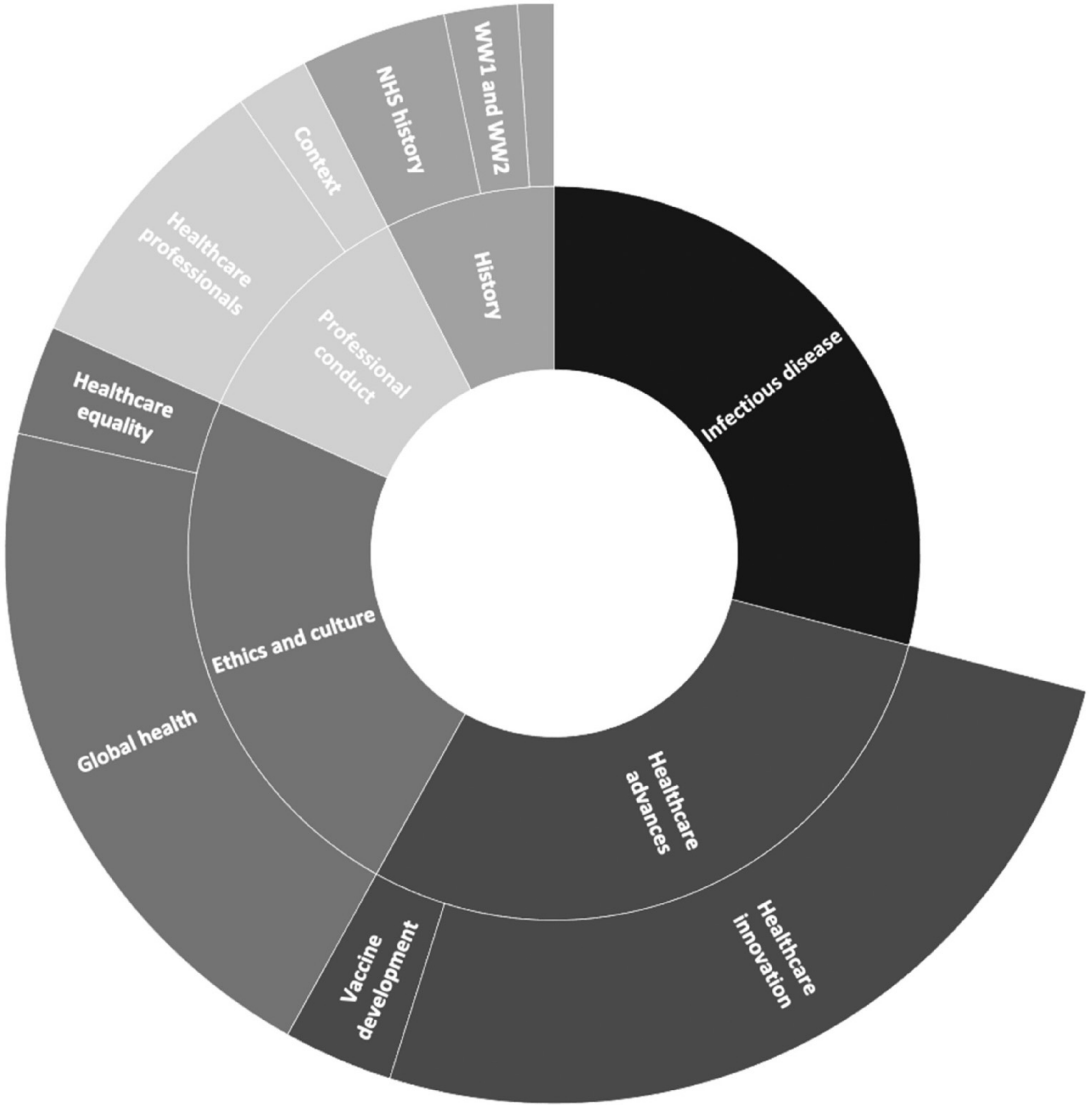
Graph displaying number of students who said yes to including additional topics in the medical school curriculum with regards to the history of medicine divided into coding categories.

52% (92/177) of students felt there was more they wanted to be included within their course while 33% (65/177) felt there wasn’t and 11% (20/117) were unsure. 94 students commented on what they felt should be taught, the emergent themes for this can be seen in Figure 6.

The majority (29% 27/94) of student's responses centred on history and in particular infectious disease history and development of medical and surgical techniques (24/94). More specifically, responses demonstrated key themes including providing more information on how pandemics have been dealt with previously and how certain diseases in the developed world have been eradicated over time. Ethics and culture was also a common theme with a desire to learn the “…specific basis for key ethno-cultural attitudes towards treatments…”, “…socioeconomic inequalities…” and “traditional medical practises…”. A further theme that emerged centred on an understanding of previous medical injustice with a desire to learn “… Guatemalan STD trials as an example of malpractice”, “…Racial bias…” and “Medicine around the world – we are not all equally developed”.

Finally, students were asked on if they had any other comments they wanted to add. A handful of students answered with responses including “*Being integrated and a natural part of the course is important…because the history of medicine provides context to modern medical practices…”*.

## Discussion

To our knowledge, this is the first known study assessing attitudes and knowledge of UK medical students on the history of Medicine in relation to historical pandemics. We present data demonstrating that overall, students displayed a positive stance to the inclusion of the history of Medicine in relation to historical pandemics in the curriculum with only 34% of respondents commenting that it was included. Knowledge on pandemics and infection control was poor - only 5.7% of students answered all questions correctly. Of the 72% of students who had a positive stance on the importance of learning about the history of Medicine, 87% of student's responses were coded into the theme “future practice”. Additionally, students were positive (79%) about the benefit of learning about the H1N1 1918 Influenza pandemic given the current COVID-19 pandemic.

While the recent COVID-19 outbreak has led to an array of questions surrounding medical education, such as the role of medical students within a pandemic and effects on medical education, it should perhaps also lead to a re-assessment of the constitution of the medical curriculum.^14,15,22^ Our extensive literature search demonstrated that there are potential benefits of teaching the history of Medicine to medical students – not only the direct knowledge gained but also in terms of context relating to historical perspectives as shown in 2012 with a history of Medicine seminar in an MD program.^25^ Arguments have been made on the role of Medical history for the doctor since at least 1904.^26^ Despite this, there is little literature assessing the role.

In 1948 Winston Churchill spoke to the commons stating “*Those who fail to learn from history are condemned to repeat it”.*^27^ Throughout COVID-19, comparisons in how the pandemic was dealt with were made to the 1918 and 2009 influenza pandemics given the significant impact on the population. Past pandemics have often led to significant changes in public health policies and healthcare systems. In 1918, the influenza pandemic led to significant improvements in disease surveillance and vaccine development.^10^ By understanding these changes, it allows decision makers to make informed choices about resource allocation and campaigns to reduce transmission such as vaccine introduction. Epidemiologists have argued that when comparing the 1918 and 2009 pandemics the earlier implementation of social distancing and school closures in 1918 had a larger effect on fewer overall cases and improved long term economic outcomes.^28^ More specifically in terms of the role of medical professionals, when comparing the 2009 pandemic to Covid-19, there are striking similarities in morbidity and the physiological effect of the viruses and the number of ICU admissions.^21^ In 2009, hospitals unfortunately had difficulty coping with the numbers of admissions, staffing levels, antiviral treatment, and supply of ventilation equipment.^29^ In 2020, unfortunately the same happened again with hospital staff struggling to get hold of vital personal protective equipment and hospitals struggling to cope with the number of admissions and the demand on ventilators.^30^ Thus, posing the question, if medical professionals had a greater understanding of previous pandemics, could we have potentially prevented erroneous decisions from being made?

More generally, within the UK, the incorporation of history of Medicine within the undergraduate curriculum is haphazard and guided by the force of interested personalities as demonstrated by our results. The situation is no different in the postgraduate context. With an American study of anaesthetic residency programs showing a low rate of inclusion of the history of anaesthesia.^31^ Even within public health domains, the process of integration of the history of medicine into the curriculum has been described as infiltrative rather than overt.^26^ The advantages of including the history of medicine of pandemics in the curriculum are clearly understood by the students we assessed with the majority of students demonstrating a positive stance on the inclusion. This fits in with a popular approach used by medical schools which focuses less on lectures and more on a humanistic and student-centred approach to guide topics which has been demonstrated to aid long-term memory formation and deepen understanding.^32-34^

This study is the first to suggest the potential wider benefit of contextualising learning within a history of medicine framework in relation to historical pandemics. Our results highlight that students are keen to learn from the past and include the history of Medicine in the curriculum, deeming that it would deepen their understanding of culturally important topics. Additionally, making mental connections is critical to our learning and context matters. Healthcare is a developing field and students need to be able to identify context and learn from this to be effective clinicians.^35^ However, students raised concerns which were rightly centred on the crowding of the curriculum. Medical degrees are re-owned for the enormous amount of content which often leaves students having to memorise facts and lacking a deeper understanding of the topic and problem solving skills.^36,37^ Stress and burnout is unfortunately common in the UK with 1/3 of doctors being affected.^38^ Medical school is demanding with students being expected to memories enormous amounts of content, cope with everyday stress and deal with traumatic and difficult situations on a not uncommon basis. The effect of not being able to cope and process crises not only has a potential detrimental effect on students health but also has been suggested to impede memory formation and performance and may play a role in reducing cognitive empathy.^39,40^ By institutions developing an understanding that factors such as stress can have a negative impact on not only students but also memory formation and reduce the likelihood of positive clinician-patient interactions and addressing these areas where possible – this can help to ensure that future doctors are greater prepared for the challenges that they may face in their careers.^41^

The effectiveness of previous teaching on history of pandemics in answering some of our knowledge-based questions suggests that a history of medicine-based pedagogy may increase interest, incorporate historical lessons, and facilitate learning on modern medical practice helping to ensure that future doctors feel better prepared for situations in their practice. This is of a high degree of relevance with society turning to previous pandemics for guidance and the similarities drawn between the current COVID-19 and the 1918 and 2009 Influenza pandemics epidemiological curves.^5-7^ As other researchers have suggested, teaching medical students positive coping mechanisms and ways to improve their quality of life during medical school can have a huge impact on preparing them for future challenges in their careers.^42,43^

### Limitations of study

Despite the findings of this study, there are several limitations including that of sample size. At the time of completing this study, there were a total of 54,00 medical students in the UK divided between 41 medical schools. The questionnaire was distributed to all UK medical schools to allow for maximum participation in our study. These results represent a sampling of 20% of medical schools within the UK and include students from both non-graduate and graduate entry programmes and at all stages in their medical degree. Thus while reduced, the sample size does demonstrate a good cross-section of the medical school cohort. This study only captures a cross sectional population at a time when pandemics are in active memory. Additionally, the data may be skewed by reporting bias with students who are more interested in the topic being more likely to answer. There was also an element of weighted reporting favouring certain medical schools thus decreasing the generalisability of our results.

### Suggestions for implementation

Recent events and students’ expectations as presented in this study, suggest the need to incorporate pandemics and pandemic management into the medical school curriculum. Our findings suggest the need to include incorporation of the development of key medical advances within learning about these advances to contextualise the intervention and therefore aid memory formation deepening mental connections. In terms of the practicalities of this, over half of students felt it would be beneficial to include in lectures versus the remaining half preferring the history of medicine such as pandemics be better incorporated into other aspects of their learning eg, case-based sessions. There may be a wider need to train medical educations in a narrative approach to teaching to better incorporate the history of medicine into the curriculum. Additionally, it may be useful for a future study to invite staff from UK medical schools who are involved in the current curriculum to also answer the survey and assess their views on curriculum development and the inclusion of the history of pandemics in the curriculum.

## Conclusions

To conclude, our evidence suggests that there are significant gaps in medical students understanding of pandemics and infection control with the majority of students displaying a positive stance to the inclusion of the history of pandemics in the medical school curriculum. Recent events and feedback from students suggest the need to incorporate pandemics and pandemic management in the curriculum with the majority of students favouring including it in lectures. Our findings indicate that such teaching may improve understanding of key concepts within infectious disease and students appreciate the value of learning about the history of pandemics. It is vital that today's medical students and therefore tomorrow's doctors are taught about such important historical events to ensure that they are as well prepared as they can be when history repeats itself.

## Supplemental Material

sj-doc-1-mde-10.1177_23821205231210629 - Supplemental material for History Repeats Itself: The Relevance of Historical Pandemics to the Medical School Curriculum Click here for additional data file. Supplemental material, sj-doc-1-mde-10.1177_23821205231210629 for History Repeats Itself: The Relevance of Historical Pandemics to the Medical School Curriculum by M Jones, S Quenby and J Odendaal in Journal of Medical Education and Curricular Development

sj-docx-2-mde-10.1177_23821205231210629 - Supplemental material for History Repeats Itself: The Relevance of Historical Pandemics to the Medical School Curriculum Click here for additional data file. Supplemental material, sj-docx-2-mde-10.1177_23821205231210629 for History Repeats Itself: The Relevance of Historical Pandemics to the Medical School Curriculum by M Jones, S Quenby and J Odendaal in Journal of Medical Education and Curricular Development
