# Supplementary material for: History Repeats Itself: The Relevance of Historical Pandemics to the Medical School Curriculum
Source: J Med Educ Curric Dev. 2023 Nov 9;10:23821205231210629. doi: 10.1177/23821205231210629 (PMC10637147; doi:10.1177/23821205231210629)
Supplement: sj-doc-1-mde-10.1177_23821205231210629 - Supplemental material for History Repeats Itself: The Relevance of Historical Pandemics to the Medical School Curriculum [file sj-doc-1-mde-10.1177_23821205231210629.doc]

STROBE checklist of items that should be included in reports of cross-sectional case-control observational studies with detailed referencing of requirements to the text of the paper.

|  | | Item No | Recommendation |
| --- | --- | --- | --- |
| **Title and abstract** | | 1 | (*a*) Indicate the study’s design with a commonly used term in the title or the abstract  Cross-sectional cohort study as stated in the Abstract on page 1 and Methods on page 6 |
|  |  |  | (*b*) Provide in the abstract an informative and balanced summary of what was done and what was found  Provided in Abstract on page 1 and 2 |
| Introduction | | | |
| Background/rationale | | 2 | Explain the scientific background and rationale for the investigation being reported  Included in the Introduction on pages 3, 4 and 5 |
| Objectives | | 3 | State specific objectives, including any prespecified hypotheses  Included in the Introduction on page 5 |
| Methods | | | |
| Study design | | 4 | Present key elements of study design early in the paper  Included in the Methods on pages 5, 6 and 7. |
| Setting | | 5 | Describe the setting, locations, and relevant dates, including periods of recruitment, exposure, follow-up, and data collection  Included in the Methods on pages 5, 6 and 7 |
| Participants | | 6 | (*a*) *Cohort study*—Give the eligibility criteria, and the sources and methods of selection of participants. Describe methods of follow-up  Included in the Methods on page 7  *Case-control study*—Give the eligibility criteria, and the sources and methods of case ascertainment and control selection. Give the rationale for the choice of cases and controls  *Cross-sectional study*—Give the eligibility criteria, and the sources and methods of selection of participants |
|  |  |  | (*b*) *Cohort study*—For matched studies, give matching criteria and number of exposed and unexposed  Not applicable.  *Case-control study*—For matched studies, give matching criteria and the number of controls per case |
| Variables | | 7 | Clearly define all outcomes, exposures, predictors, potential confounders, and effect modifiers. Give diagnostic criteria, if applicable  Included in the Methods on pages 6, 7 and 8. |
| Data sources/ measurement | | 8* | For each variable of interest, give sources of data and details of methods of assessment (measurement). Describe comparability of assessment methods if there is more than one group  Included in the Patients and Methods on pages 7 and 8 |
| Bias | | 9 | Describe any efforts to address potential sources of bias  Addressed in the limitations paragraph on page 24. |
| Study size | | 10 | Explain how the study size was arrived at  Included in the Methods on page 7 |
| Quantitative variables | | 11 | Explain how quantitative variables were handled in the analyses. If applicable, describe which groupings were chosen and why  Included in the Patients and Methods on pages 7 and 8 |
| Statistical methods | | 12 | (*a*) Describe all statistical methods, including those used to control for confounding  Included in the Patients and Methods on pages 7 and 8 |
|  |  |  | (*b*) Describe any methods used to examine subgroups and interactions  Included in the Patients and Methods on pages 7 and 8. |
|  |  |  | (*c*) Explain how missing data were addressed  Included in the Methods on page 7 |
|  |  |  | (*d*) *Cohort study*—If applicable, explain how loss to follow-up was addressed  Not applicable  *Case-control study*—If applicable, explain how matching of cases and controls was addressed  *Cross-sectional study*—If applicable, describe analytical methods taking account of sampling strategy |
|  |  |  | (*e*) Describe any sensitivity analyses Not applicable. |
| Results | | | |
| Participants | 13* | (a) Report numbers of individuals at each stage of study—eg numbers potentially eligible, examined for eligibility, confirmed eligible, included in the study, completing follow-up, and analysed  Included in the Results on pages 9 and 10. | |
|  |  | (b) Give reasons for non-participation at each stage  Included in the results on pages at each stage on pages 9-19 | |
|  |  | (c) Consider use of a flow diagram  Included in the Results on page 10. | |
| Descriptive data | 14* | (a) Give characteristics of study participants (eg demographic, clinical, social) and information on exposures and potential confounders  Included in the Results on page 9. | |
|  |  | (b) Indicate number of participants with missing data for each variable of interest  Included in the results throughout each stage on pages 9-19. | |
|  |  | (c) *Cohort study*—Summarise follow-up time (eg, average and total amount)  Included in the results on page 9. | |
| Outcome data | 15* | *Cohort study*—Report numbers of outcome events or summary measures over time  Included in the results on pages 9-19. | |
|  |  | *Case-control study—*Report numbers in each exposure category, or summary measures of exposure | |
|  |  | *Cross-sectional study—*Report numbers of outcome events or summary measures | |
| Main results | 16 | (*a*) Give unadjusted estimates and, if applicable, confounder-adjusted estimates and their precision (eg, 95% confidence interval). Make clear which confounders were adjusted for and why they were included  Not applicable. | |
|  |  | (*b*) Report category boundaries when continuous variables were categorized  Not applicable | |
|  |  | (*c*) If relevant, consider translating estimates of relative risk into absolute risk for a meaningful time period.  Not applicable. | |
| Other analyses | 17 | Report other analyses done—eg analyses of subgroups and interactions, and sensitivity analyses  Included in the Results on pages 13-16. | |
| Discussion | | | |
| Key results | 18 | Summarise key results with reference to study objectives  Included in the Discussion on page 21. | |
| Limitations | 19 | Discuss limitations of the study, taking into account sources of potential bias or imprecision. Discuss both direction and magnitude of any potential bias  Included in the Discussion on page 24 | |
| Interpretation | 20 | Give a cautious overall interpretation of results considering objectives, limitations, multiplicity of analyses, results from similar studies, and other relevant evidence  Included in the Discussion on pages 21 to 25 | |
| Generalisability | 21 | Discuss the generalisability (external validity) of the study results  Included in the Discussion on page 25. | |
| Other information | | | |
| Funding | 22 | Give the source of funding and the role of the funders for the present study and, if applicable, for the original study on which the present article is based  Provided in the declarations on page 26. | |
