# Supplementary material for: History Repeats Itself: The Relevance of Historical Pandemics to the Medical School Curriculum
Source: J Med Educ Curric Dev. 2023 Nov 9;10:23821205231210629. doi: 10.1177/23821205231210629 (PMC10637147; doi:10.1177/23821205231210629)
Supplement: sj-docx-2-mde-10.1177_23821205231210629 - Supplemental material for History Repeats Itself: The Relevance of Historical Pandemics to the Medical School Curriculum [file sj-docx-2-mde-10.1177_23821205231210629.docx]

Supplementary table 1: Questionnaire

|  | **Question Number** | **Question** | **Type of response** |
| --- | --- | --- | --- |
| **Demographics of medical students** | 1 | What medical school do you attend? | Drop down list of all UK medical schools |
|  | 2 | Are you on an accelerated 4-year Graduate Entry Programme? | Yes/No |
|  | 3 | What is your current year of study | Options ranging from years 1-6 |
|  |  | How long is your course? | Options: 4-6 years / other |
| **Knowledge about the H1N1 Pandemic and Infection Control** | 4 | Have you had any teaching on historical pandemics at medical school?  If no – would you feel it would be beneficial | Yes/No  Free text |
|  | 5 | Have you had any teaching about the H1N1 1918 Influenza pandemic? | Yes/No |
|  |  | If no – would you feel it would be beneficial | Free text |
|  | 6 | What is the difference between an epidemic and a pandemic?   1. A pandemic affects the world whilst an epidemic affects one country or region 2. A pandemic affects one country or region whilst an epidemic affects the world | 2 multiple choice options |
|  | 7 | Select all correct answers for how influenza can spread   1. Close contact with infected person 2. Sexual contact 3. Blood transfusions 4. Coughs and sneezes from an infected person 5. Contact with infected animals | 5 multiple choice options |
|  | 8 | Select all correct answers for how an influenza pandemic can be prevented   1. Nothing – there is no prevention 2. Social distancing 3. Antibiotics 4. Covering mouth and nose when coughing and sneezing 5. Hand washing 6. Staying home 7. Wearing protective clothing in public places 8. Antiviral drugs 9. Vitamins and herbal supplements 10. Vaccinations | 10 multiple choice options |
|  | 9 | Select all correct answers for how influenza can be treated   1. Nothing – there is no treatment 2. Bed rest 3. Antibiotics 4. Antiviral drugs 5. Vitamins and herbal supplements | 5 multiple choice options |
| **History of Medicine** | 10 | Is history of Medicine taught at your medical school? | Yes/No |
|  |  | If yes – how is it taught? | Free text |
|  |  | If yes – what is included | Free text |
|  | 11 | The history of medicine is important to learn about at medical school?   1. Strongly agree 2. Slightly agree 3. Neither agree nor disagree 4. Slightly disagree 5. Strongly disagree | Likert scale with 5 options |
|  |  | If you answered in agreement, why? | Free text |
|  | 12 | It would be beneficial to know more about the H1N1 pandemic, given the current circumstances with COVID-19   1. Strongly agree 2. Slightly agree 3. Neither agree nor disagree 4. Slightly disagree 5. Strongly disagree | Likert scale with 5 options |
|  | 13 | Are there any other aspects of the history of Medicine that you would like to be included in your course? | Free-text |
|  | 14 | Any other comments |  |
